# Predictive value of models based on MRI radiomics and clinical indicators for lymphovascular space invasion in endometrial cancer

**DOI:** 10.1186/s12885-025-14217-6

**Published:** 2025-04-28

**Authors:** Wenwen Ma, Weijing Meng, Jinfeng Yin, Jie Liang, Xizhen Wang, Jingang Liu, Fuyan Shi

**Affiliations:** 1 Affiliated Hospital of Shandong Second Medical University, Weifang, 261053 China; 2 School of Public Health, Shandong Second Medical University, Weifang, 261053 China; 3 School of Medical Imaging, Shandong Second Medical University, Weifang, 261053 China; 4 Weifang People‘s Hospital, Shandong Second Medical University, Weifang, 261053 China

**Keywords:** Endometrial cancer, Radiomics, Clinical indicators, Lymphovascular space invasion, Magnetic resonance imaging

## Abstract

**Background:**

Lymphovascular space invasion (LVSI), a prognostic indicator closely associated with tumour invasiveness, lymph node metastasis risk, and recurrence rate, is crucial in endometrial cancer (EC) staging; however, LVSI is currently diagnosed via postoperative pathology, highlighting the need for non-invasive diagnostic methods. This study aimed to investigate the predictive value of intratumoural and peritumoral magnetic resonance imaging (MRI) multiparametric radiomics combined with clinical indicators of LVSI in EC.

**Methods:**

This retrospective analysis included 310 patients with EC who underwent preoperative MRI examinations at the Affiliated Hospital of Shandong Second Medical University (Centre A) and the First Clinical Medical College of Shandong Second Medical University (Centre B). The patients were divided into training (Centre A) and validation (Centre B) sets. Clinically independent risk factors and intratumoural and peritumoural radiomic characteristics were screened. Five models were constructed: clinical, peritumoural radiomics, intratumoural radiomics, combined intratumoural and peritumoural radiomics, and combined clinical, intratumoural, and peritumoural radiomics. A nomogram was constructed based on the optimal model. The diagnostic efficacy of the five models was evaluated using area under the curve. The accuracy of the model was evaluated using calibration curves, and the clinical value of the model was analysed using decision curve analysis.

**Results:**

Logistic regression analysis identified CA125 and tumour length as independent risk factors for LVSI in EC. Among the five models, the combined clinical + intratumoural + peritumoural radiomics model performed slightly better than the other four models, with area under the curve values of 0.870 (95% CI: 0.821–0.919) for the training set and 0.818 (95% CI: 0.731–0.905) for the validation set. The calibration curve showed good consistency, and decision curve analysis suggested that the model had good clinical benefits.

**Conclusion:**

The combined clinical + intratumoural + peritumoural radiomics model based on clinical indicators and intratumoural and peritumoural multi-parametric MRI radiomics features demonstrated good diagnostic efficacy. This model provides a theoretical basis for preoperative evaluation of LVSI in EC.

## Background

Endometrial cancer (EC) is one of the most common gynaecological cancers worldwide and the second most common gynaecological cancer in China, with both its incidence and mortality rates increasing annually [1–3]. The primary treatment for early stage EC involves the surgical removal of the uterus and its appendages, which has an overall survival rate of approximately 90% [4]. Therefore, early detection and timely treatment are crucial for improving patient prognosis.

The presence of malignant tumours in the lymphatic or vascular areas of the myometrium is referred to as lymphovascular space invasion (LVSI) [5]. LVSI is a critical prognostic indicator closely associated with tumour invasiveness, lymph node metastasis risk, and recurrence rate, and has great significance in cancer staging, with the International Federation of Gynaecology and Obstetrics (FIGO) 2023 staging system using it as a key factor in classification [5, 6]. The presence of LVSI typically indicates a poor prognosis. Consequently, the accurate preoperative prediction of LVSI is essential for tailoring personalised surgical plans and adjusting treatment strategies. However, LVSI can only be diagnosed via postoperative pathology, which poses significant challenges in clinical practice.

Magnetic resonance imaging (MRI) is a common preoperative examination modality for EC that accurately assesses the extent of local lesions and disseminated extrauterine malignancies [2, 7, 8]. However, the use of MRI to diagnose LVSI preoperatively remains challenging. Studies have reported that the prediction accuracy rate of MRI in the preoperative assessment of disease stage is only 47.2% [9], highlighting the urgent need for an effective tool to assess LVSI status preoperatively. In this regard, radiomics analysis can extract tumour features from high-throughput images that are difficult to observe with the naked eye, characterise tumour heterogeneity and microenvironment information, and thus, show great potential for tumour detection, diagnosis, and prognostic evaluation [10–13]. Some studies have demonstrated that models established using MRI radiomics have good predictive performance for the assessment of LVSI and lymph node metastasis in EC [14–16]. However, these studies were limited to the intratumoural region and ignored the tumour microenvironment. Recent studies have suggested that the microenvironment surrounding tumours can help understand the clinical behaviour of tumour lesions [17–19].

Therefore, this study aimed to compare the clinical application value of clinical, intratumoural, and peritumoural MRI radiomics in predicting LVSI status using clinical indicators and imaging data from patients with EC, providing more effective guidance for clinical treatment and improving patient prognosis.

## Methods

### Participants

**Fig. 1:**
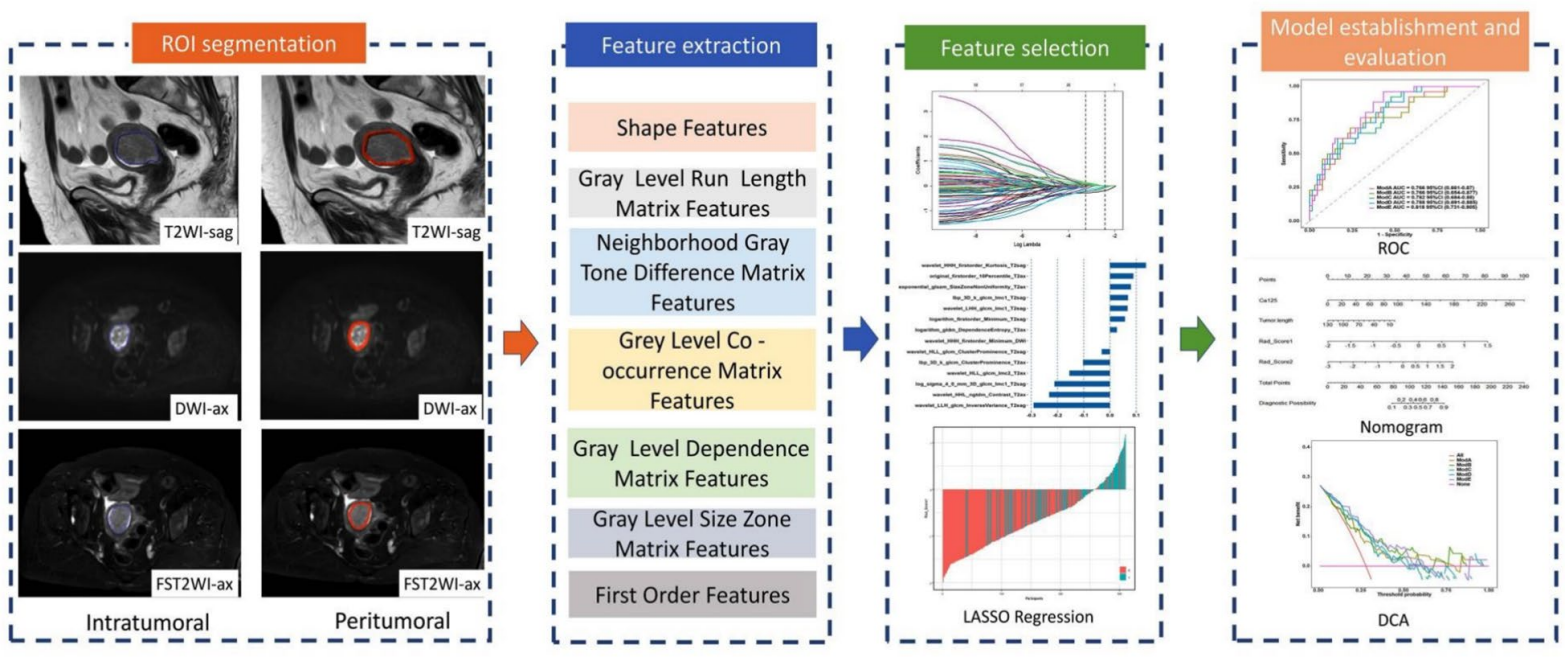
Study Framework

**Fig. 2:**
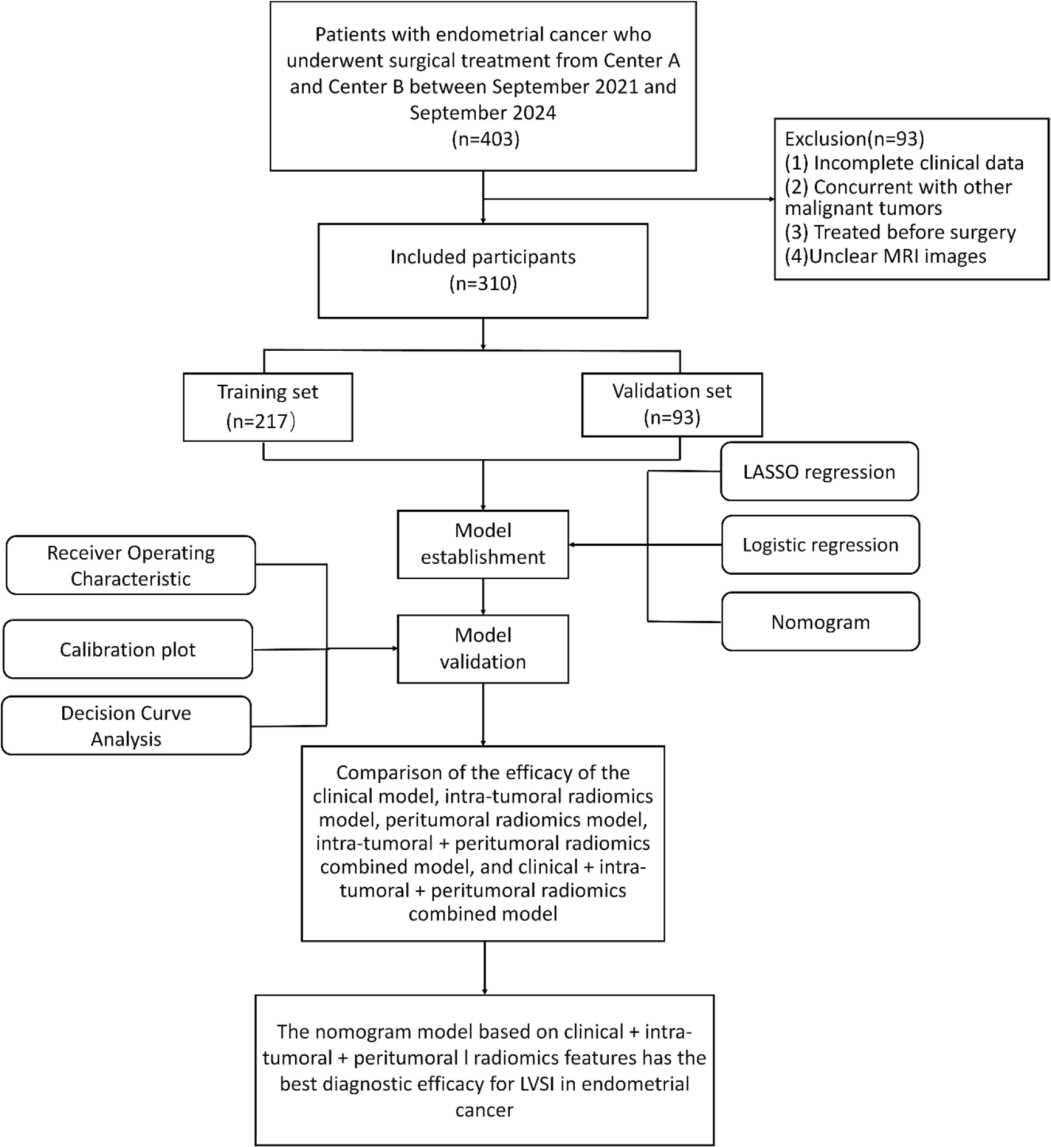
Research pathway

The MRI images of 403 patients with EC who underwent surgical treatment at Shandong Second Medical University Affiliated Hospital (Centre A) and Shandong Second Medical University First Clinical Medical College (Centre B) between September 2021 and September 2024 were collected. The inclusion criteria were as follows: (1) patients first diagnosed with EC via pathology who underwent radical hysterectomy; (2) routine pelvic MRI non-contrast examination performed within two weeks before surgery; and (3) clear MRI images and complete pathological information. The exclusion criteria were as follows: (1) incomplete clinical data; (2) other concurrent malignant diseases; (3) treatment before surgery; and (4) unclear MRI images. The pathological evaluation criteria for LVSI status were as follows: infiltration of tumour cells in the lymphatic vessels or tumour cells invading the blood vessel wall, and formation of small cancer emboli within the blood vessel wall. The clinical data collected for each patient included age, tumour length, FIGO stage (obtained from the MRI diagnostic report), carbohydrate antigen 125 (CA125) level, hypertension, diabetes, and body mass index (BMI). The framework and research pathways of this study are shown in Figs. 1 and 2, respectively.

This study adhered to the ethical guidelines of the Declaration of Helsinki and was approved by the Medical Ethics Committee of Shandong Second Medical University (IRB number: SDSMU2025YX003).

### Instruments and methods

A Philips 1.5 T (A centre)/3.0 T (B centre) MRI system and an abdominal 8-channel phased array coil were used for imaging. Prior to examination, the patients were instructed to empty their bladders. During the examination, patients were placed in the supine position, and the abdomen and pelvis were fixed with an abdominal band to reduce motion artefacts and calm breathing. The MRI sequences included pelvic axial T1-weighted imaging (T1 WI), axial T2-weighted imaging (T2 WI), axial fat-suppressed T2-weighted imaging (FS-T2 WI), axial diffusion-weighted imaging (DWI), and sagittal T2-weighted imaging (T2 WI). The scanning parameter settings were as follows: Repetition Time (TR), 3000–4000 ms; Echo Time (TE), 85–100 ms; layer thickness, 3–4 mm; layer spacing, 0.5 ~ 1 mm, maximum field of view (FOV), 315 × 400 mm; and matrix, 256 × 256 or 512 × 512. If the lesion was large, the scan widens.

### Image segmentation

Images from the axial DWI, axial fat-suppressed T2 WI, and sagittal T2 WI sequences were imported into the Deepwise Multimodal Research Platform version 2.5.1 (https://keyan.deepwise.com; Hangzhou Deepwise & League of PHD Technology Co., Ltd., Hangzhou, Zhejiang, China) for each patient. A deputy chief physician from the Department of Imaging delineated regions of interest (ROIs) for the intratumoural regions in each of the three sequences to ensure the reliability and replicability of the data. The ROI was drawn layer-by-layer to avoid tumour haemorrhage and necrosis. Another deputy chief physician randomly selected images from 30 patients to delineate the ROI for the intratumoural regions. The peritumoural ROI was automatically generated by a 3-mm outward expansion from the intratumoural ROI. The intraclass correlation coefficient (ICC) was calculated to evaluate and ensure consistency.

### Feature extraction and filtering

The Deepwise Multimodal Research Platform version 2.5.1 was used to normalise and extract features from the intratumoural and peritumoural ROIs for each patient. The extracted features included shape, grey level run-length matrix, neighbourhood grey tone difference matrix, grey-level co-occurrence matrix, grey-level dependence matrix, grey-level size zone matrix, and first-order features. The radiomics features extracted from the ROIs of 30 randomly selected patients were first subjected to ICC analysis to ensure data consistency and reliability. Features with an ICC value ≥ 0.9 were selected for subsequent analysis due to their stability. Subsequently, a correlation analysis was performed to filter out features with a correlation coefficient ≤ 0.7. Finally, the Least Absolute Shrinkage and Selection Operator (LASSO) algorithm was employed to reduce dimensionality, and the most predictive radiomics features for the intratumoural and peritumoural regions were selected, followed by calculation of the radiomics score (Rad_Score).

### Model establishment and evaluation

The clinical indicators were analysed using univariate logistic regression analysis. Indicators with statistical significance were subjected to multivariate logistic regression analysis to identify the independent risk factors. The significant independent risk factors identified using logistic regression analysis, along with the intratumoural and peritumoural imaging features selected by LASSO regression, were used to establish the clinical, peritumoural radiomics, intratumoural radiomics, combined intratumoural + peritumoural radiomics, and combined clinical + intratumoural + peritumoural radiomics models. A nomogram was constructed based on the optimal model. Model performance was evaluated, and their clinical values were analysed. Diagnostic efficacy of the models was assessed using the area under the receiver operating characteristic curve (AUC). Model accuracy was evaluated using calibration plots, and clinical effectiveness was assessed using decision curve analysis (DCA).

### Statistical analysis

Statistical analyses and plotting were conducted using R software (version 4.2.1). Categorical variables are expressed as frequencies and percentages and were compared using the c^2^ test or Fisher's exact test. Continuous variables are presented as mean ± standard deviation and were compared using the t-test or Mann–Whitney U test. Baseline description and difference analysis were performed using the"CBCgrps"package. LASSO regression was conducted using the"glmnet"package, while multivariate Logistic regression was performed using the"glm"package. The nomogram was created using the"rms"package, the ROC curve was plotted using the pROC package, and calibration curves were generated using both the"rms"and"riskregression"packages. The DCA was conducted using the"rmda"package. All statistical tests were two-sided, and P < 0.05 was considered statistically significant.

## Results

### Patient characteristics

Based on the inclusion and exclusion criteria, 310 patients were included in this study. Among them, 217 patients from Centre A were assigned to the training set (75 with positive LVSI and 142 with negative LVSI), and 93 patients from Centre B were assigned to the validation set (26 with positive LVSI and 67 with negative LVSI). The basic patient characteristics are summarised in Table 1.

**Table 1:** Basic patient characteristics

Variables	Total (n = 310)	No LVSI (n = 209)	LVSI (n = 101)	*P*	Test (n = 93)	Train (n = 217)	*P*
Age, year (Mean ± SD)	57.27 ± 9.29	56.78 ± 9.73	58.29 ± 8.26	0.156	58.59 ± 9.40	56.70 ± 9.21	0.104
CA125, u/ml (Mean ± SD)	37.08 ± 50.58	22.44 ± 23.65	67.38 ± 73.26	< 0.001	27.35 ± 41.62	41.25 ± 53.52	0.014
FIGO, n (%)				0.096			0.684
I	273 (88)	180 (86)	93 (92)		84 (90)	189 (87)	
II	27 (9)	23 (11)	4 (4)		6 (6)	21 (10)	
III	10 (3)	6 (3)	4 (4)		3 (3)	7 (3)	
Tumor length, mm (Mean ± SD)	34.11 ± 18.88	30.45 ± 16.62	41.7 ± 21	< 0.001	29.01 ± 17.58	36.3 ± 19.03	0.001
Hypertension, n (%)				0.951			0.080
No	188 (61)	126 (60)	62 (61)		49 (53)	139 (64)	
Yes	122 (39)	83 (40)	39 (39)		44 (47)	78 (36)	
Diabetes, n (%)				0.482			0.487
No	251 (81)	172 (82)	79 (78)		78 (84)	173 (80)	
Yes	59 (19)	37 (18)	22 (22)		15 (16)	44 (20)	
BMI, kg/m^2^ (Mean ± SD)	26.71 ± 3.91	26.97 ± 3.92	26.18 ± 3.87	0.096	26.65 ± 3.7	26.74 ± 4.01	0.849

*FIGO* staging is derived from image reports, *BMI* Body Mass Index

### Radiomics feature selection results

**Fig. 3:**
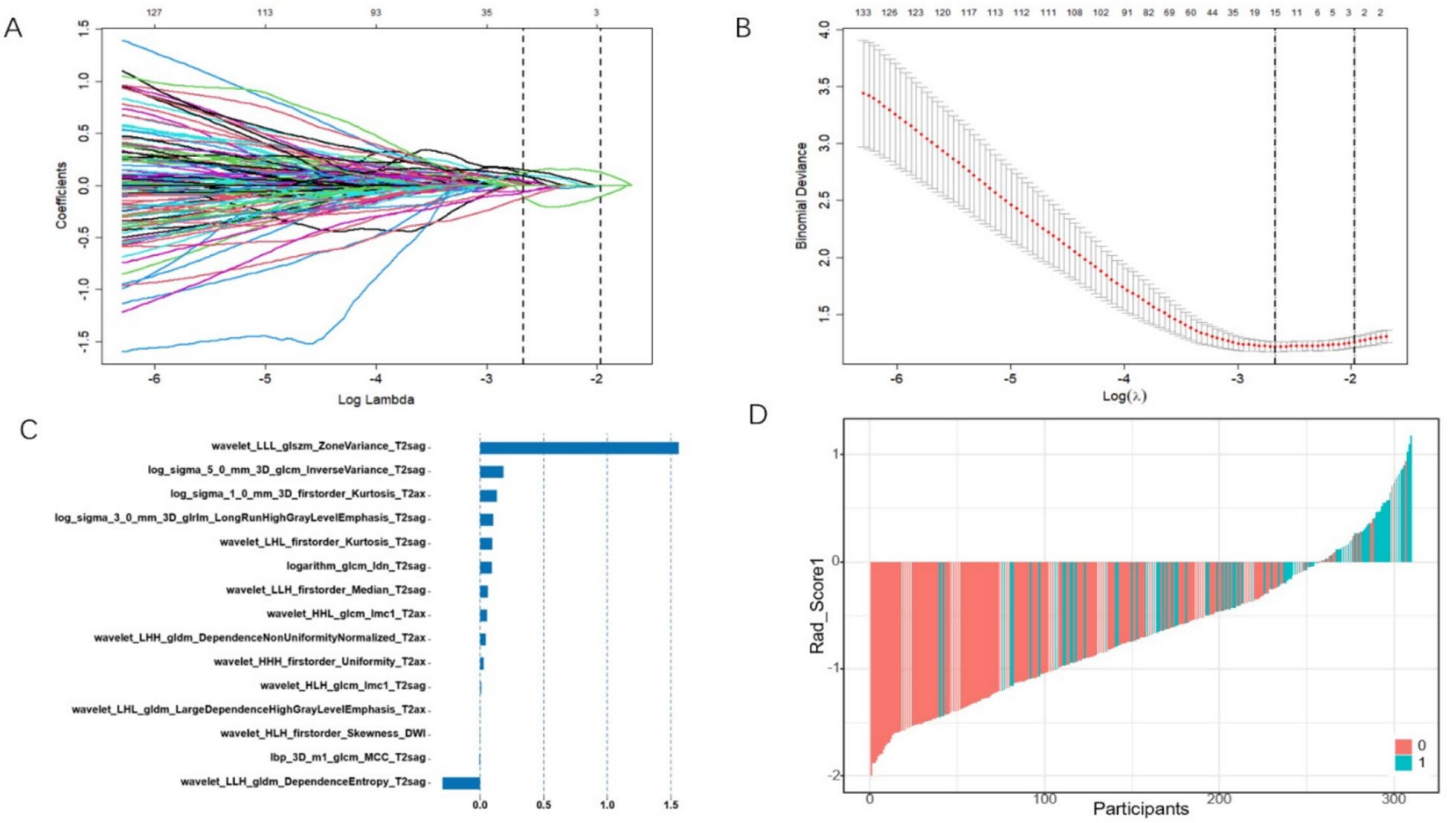
Intratumorial Radiomics feature selection using Lasso Regression. **A** The LASSO regression path diagram; **B** The plot of the important features screened by the ten-fold cross validation method. The important features were selected using lambda.min as the criterion. **C** The 15 important features selected by LASSO regression and their weight chart. **D** The waterfall chart for all participants

**Fig. 4:**
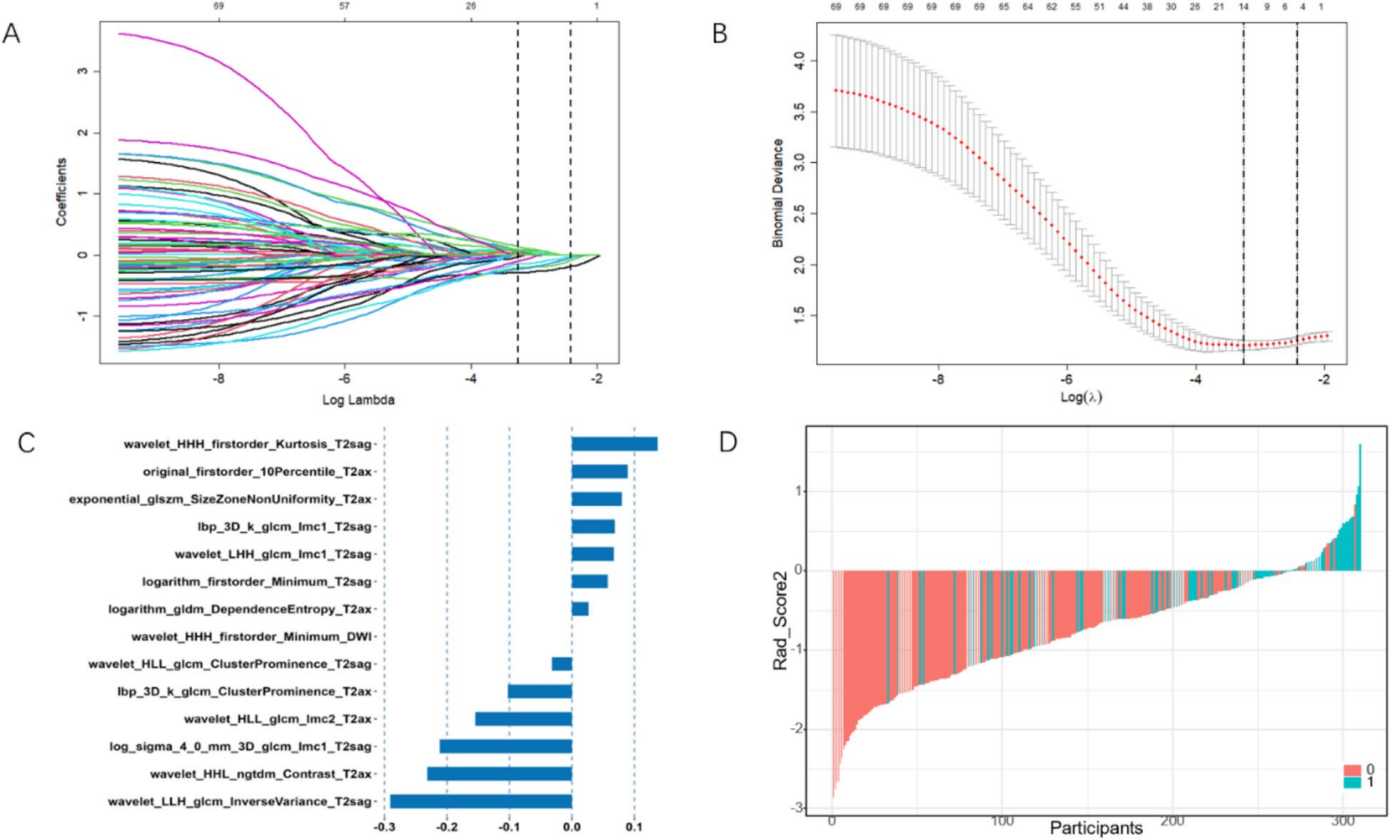
Peritumoral Radiomics feature selection using Lasso Regression. **A** The LASSO regression path diagram; **B** The plot of the important features screened by the ten-fold cross validation method. The important features were selected using lambda.min as the criterion. **C** The 14 important features selected by LASSO regression and their weight chart. **D** The waterfall chart for all participants

In this study, 6455 features were extracted from the intratumoural and peritumoural regions. After internal consistency testing using the ICC, feature correlation analysis, and LASSO regression dimensionality reduction, 15 and 14 features were selected from the intratumoral and peritumoural regions, respectively. The selected intratumoural and peritumoural radiomics features are shown in Tables 2 and 3, respectively. The variable shrinkage and cross-validation processes for the intratumoural region are illustrated in the LASSO regression plots shown in Figs. 3A and 3B. The 15 selected intratumoural features and their corresponding weights are shown in Fig. 3C, and a waterfall plot for all participants is shown in Fig. 3D. The variable shrinkage and cross-validation processes for the peritumoural region are illustrated in the LASSO regression plots shown in Figs. 4A and 4B. The 14 selected important peritumoural features and their corresponding weights are shown in Fig. 4C, and a waterfall plot for all participants is shown in Fig. 4D. The intratumoural and peritumoural radiomics scores (Rad_Score) for each patient were calculated using the following formula: Rad_Score = Σ (feature value * feature coefficient) + b0 (intercept).

**Table 2:** Results of intratumoral radiomics feature screening

Numbering	Feature	Coefficient
1	squareroot_firstorder_Skewness_T2 sag	0.14729117
2	log_sigma_5_0_mm_3D_gldm_DependenceVariance_T2 sag	0.12502147
3	log_sigma_2_0_mm_3D_firstorder_Skewness_T2ax	0.11919266
4	log_sigma_1_0_mm_3D_glcm_InverseVariance_T2 sag	0.11878343
5	log_sigma_1_0_mm_3D_glrlm_RunLengthNonUniformityNormalized_T2 sag	0.11249461
6	log_sigma_3_0_mm_3D_gldm_LargeDependenceHighGrayLevelEmphasis_T2ax	0.09116579
7	wavelet_LHH_ngtdm_Busyness_T2ax	0.09110913
8	wavelet_HLL_glcm_MCC_T2ax	0.06597751
9	log_sigma_5_0_mm_3D_glcm_DifferenceEntropy_T2 sag	0.05677993
10	square_glcm_InverseVariance_DWI	0.04846720
11	squareroot_firstorder_Kurtosis_T2 sag	0.04035398
12	lbp_2D_glszm_SizeZoneNonUniformityNormalized_T2ax	0.02790663
13	lbp_2D_glszm_SmallAreaHighGrayLevelEmphasis_T2ax	0.01992961
14	log_sigma_1_0_mm_3D_glcm_Idm_T2 sag	0.01208342
15	lbp_3D_m2_firstorder_Maximum_T2ax	0.01089080

**Table 3:** Results of peritumoral radiomics feature screening

Numbering	Feature	Coefficient
1	wavelet_LLH_glcm_InverseVariance_T2 sag	0.2905970542
2	wavelet_HHL_ngtdm_Contrast_T2ax	0.2315941418
3	log_sigma_4_0_mm_3D_glcm_Imc1_T2 sag	0.2112325945
4	wavelet_HLL_glcm_Imc2_T2ax	0.1544878177
5	wavelet_HHH_firstorder_Kurtosis_T2 sag	0.1369438683
6	lbp_3D_k_glcm_ClusterProminence_T2ax	0.1027404286
7	original_firstorder_10Percentile_T2ax	0.0892277062
8	exponential_glszm_SizeZoneNonUniformity_T2ax	0.0796758980
9	lbp_3D_k_glcm_Imc1_T2 sag	0.0687273533
10	wavelet_LHH_glcm_Imc1_T2 sag	0.0669025532
11	logarithm_firstorder_Minimum_T2 sag	0.0573108126
12	wavelet_HLL_glcm_ClusterProminence_T2 sag	0.0319443867
13	logarithm_gldm_DependenceEntropy_T2ax	0.0264802463
14	wavelet_HHH_firstorder_Minimum_DWI	0.0001748998

### Clinical Feature Selection Results

The clinical indicators age, CA125 level, tumour length, hypertension, diabetes, and BMI were first subjected to univariate logistic regression analysis. CA125 level, FIGO stage, tumour length, and BMI, were significant and included in a multivariate logistic regression analysis using the backward selection method. Ultimately, CA125 levels and tumour length were found to be statistically significant, indicating that CA125 levels and tumour length are independent risk factors for LVSI in EC. Table 4 presents the results of the study.

**Table 4:** Results of univariate and multivariate logistic regression analyses of clinical indicators

Variables	Univariate logistic analysis				Multivariate logistic analysis			
	**B**	**SE**	**OR (95%CI)**	***P***	**B**	**SE**	**OR (95%CI)**	***P***
Age (year)	0.028	0.016	1.028 (0.997–1.062)	0.083				
Ca125 (µ/mL)	0.032	0.007	1.032 (1.020–1.047)	< 0.001	0.028	0.006	1.028 (1.016–1.042)	< 0.001
FIGO II	−2.488	1.036	0.083 (0.005–0.412)	0.016	−1.803	1.047	0.164 (0.008–0.847)	0.085
FIGO III	0.220	0.778	1.246 (0.240–5.810)	0.777	−0.420	1.201	0.657 (0.035–5.456)	0.727
Tumor length (mm)	0.027	0.008	1.027 (1.012–1.044)	0.001	0.019	0.009	1.018 (1.001–1.038)	0.039
Hypertension	−0.085	0.299	0.918 (0.508–1.642)	0.776				
Diabetes	0.343	0.347	1.409 (0.706–2.768)	0.323				
BMI (kg/m^2^)	−0.098	0.040	0.906 (0.836–0.977)	0.013	−0.051	0.044	0.949 (0.869–1.032)	0.237

### Model Construction and Validation Results

**Fig. 5:**
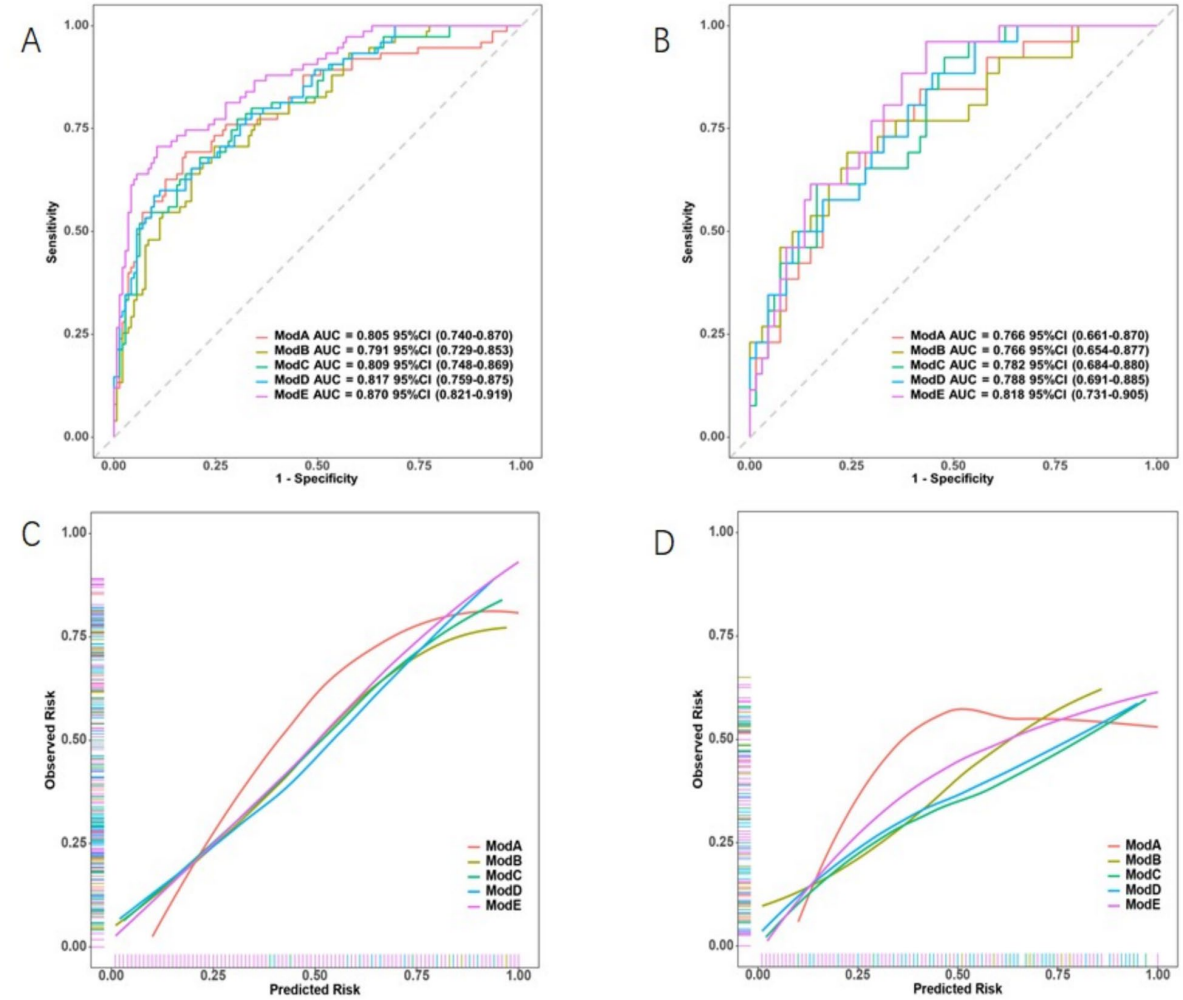
Discriminative power and accuracy of the prediction model. (**A**) and (**B**) show the receiver operating curves of the model in the training and validation sets, respectively, and (**C**) and (**D**) show the calibration curves of the model in the training and validation sets, respectively

**Fig. 6:**
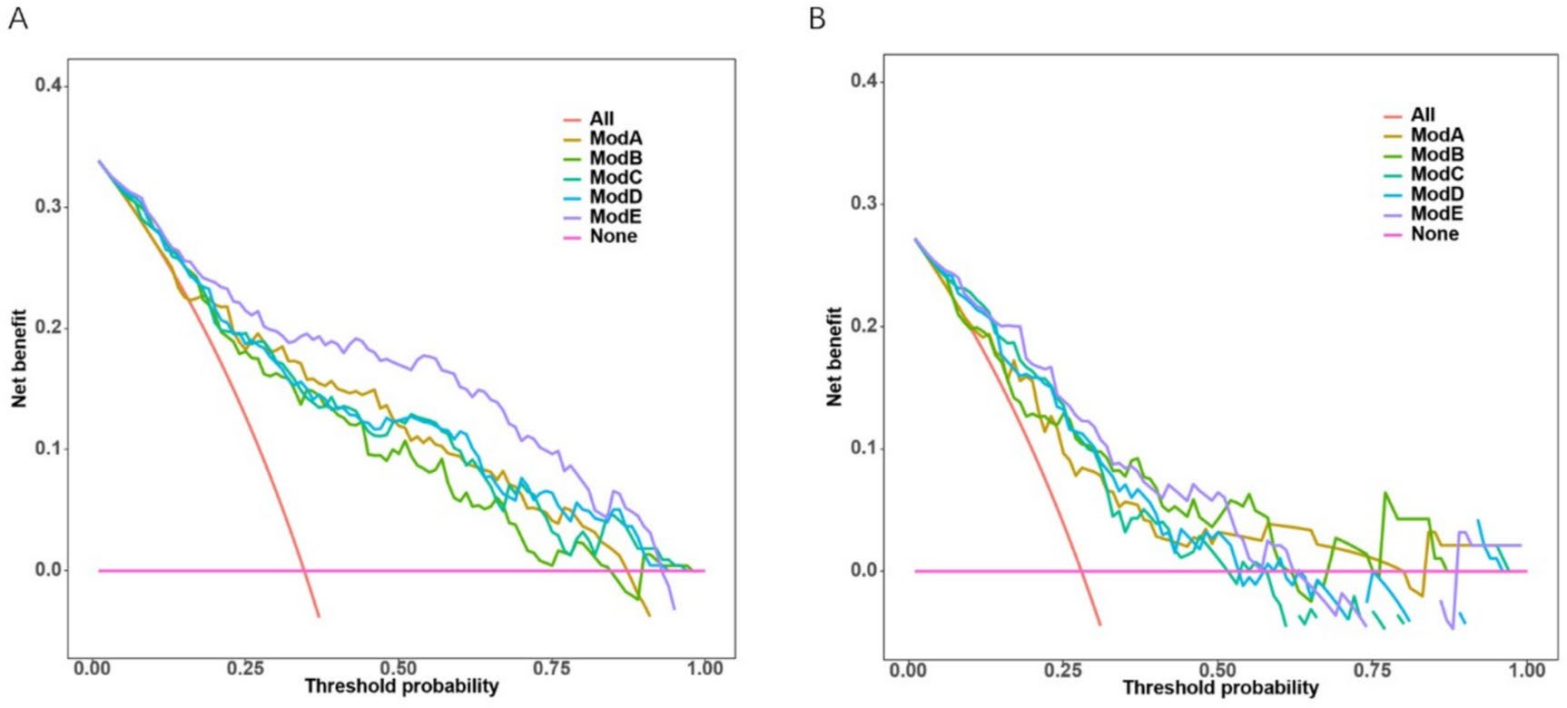
Discriminative power and accuracy of the prediction model. (**A**) and (**B**) show the clinical decision curves of the model for the training and validation sets, respectively

**Fig. 7:**
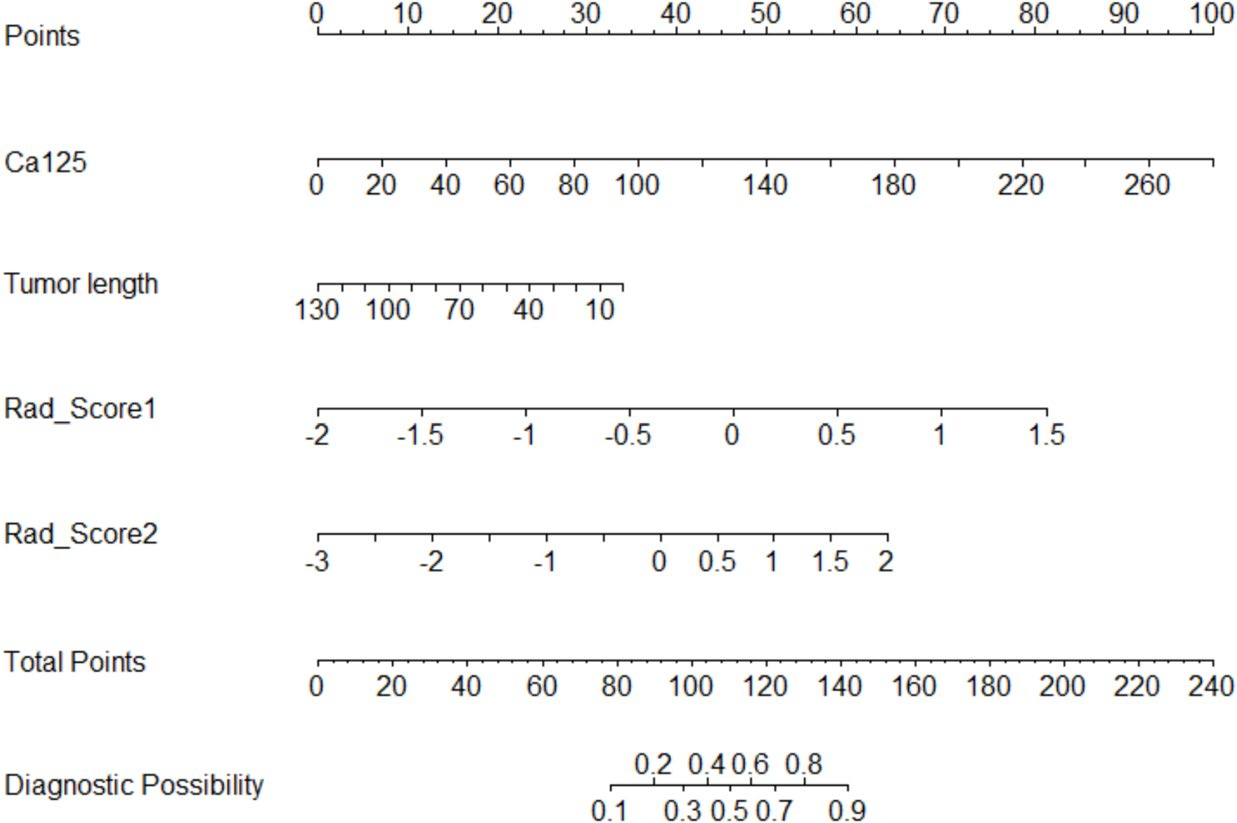
Nomogram of ModE

The clinical model was established based on CA125 levels and tumour length; the intratumoural radiomics model was established based on Rad_Score1; the peritumoural radiomics model was established based on Rad_Score2; the intratumoural + peritumoural radiomics combined model was established based on Rad_Score1 and Rad_Score2; and the clinical + intratumoural + peritumoural radiomics combined model was established based on CA125, tumour length, Rad_Score1, and Rad_Score2. The performances of the five models on the training and validation sets are presented in Tables 5 and 6, respectively. Among them, the clinical + intratumoural + peritumoural radiomics combined model performed slightly better than the other four models, with an AUC of 0.870 (95% CI: 0.821–0.919) in the training set and 0.818 (95% CI: 0.731–0.905) in the validation set. The DeLong test for the AUC values of the optimal model and other models are presented in Table 7. The ROC curves of the five models in the training and validation sets are shown in Fig. 5A and Fig. 5B, respectively. Calibration curves showed good consistency between the predicted and actual probabilities of the models (Fig. 5C and 5D). Decision curve analysis suggested that the combined clinical + intratumoural + peritumoural radiomics model could achieve a greater benefit in clinical decision-making (Fig. 6A and Fig. 6B). Subsequently, a nomogram was constructed for the combined clinical, intratumoural, and peritumoural radiomics model (Fig. 7). The nomogram intuitively predicted the risk of developing LVSI in EC by locating the corresponding points on the nomogram based on the specific values of CA125, tumour length, Rad_Score1, and Rad_Score2 for each patient and summing the points for each indicator.

**Table 5:** Comparison of five models in the training set

Model	AUC(95%CI)	ACC(95%CI)	SEN	SPE	PPV	NPV
Clinical model (ModA)	0.805 (0.740–0.870)	0.779 (0.777–0.780)	0.693	0.824	0.675	0.836
Peritumoral radiomics model (ModB)	0.791 (0.729–0.853)	0.737 (0.736–0.739)	0.707	0.754	0.602	0.829
Intratumoral radiomics model (ModC)	0.809 (0.748–0.869)	0.724 (0.722–0.725)	0.773	0.697	0.574	0.853
Intratumoral + peritumoral radiomics model (ModD)	0.817 (0.759–0.875)	0.793 (0.791–0.794)	0.587	0.901	0.759	0.805
Clinical + intratumoral + peritumoral radiomics model (ModE)	0.870 (0.821–0.919)	0.829 (0.828–0.831)	0.707	0.894	0.779	0.852

*ACC* Accuracy, *SEN* Sensitivity, *SPE* Specificity, *PPV* Positive Predictive Value, *NPV* Negative Predictive Value

**Table 6:** Comparison of five models in the validation set

Model	AUC(95%CI)	ACC(95%CI)	SEN	SPE	PPV	NPV
Clinical model (ModA)	0.766 (0.661–0.870)	0.699 (0.694–0.703)	0.769	0.672	0.476	0.882
Peritumoral radiomics model (ModB)	0.766 (0.654–0.877)	0.742 (0.738–0.746)	0.692	0.761	0.529	0.864
Intratumoral radiomics model (ModC)	0.782 (0.684–0.880)	0.774 (0.771–0.778)	0.615	0.836	0.593	0.848
Intratumoral + peritumoral radiomics model (ModD)	0.788 (0.691–0.885)	0.645 (0.640–0.650)	0.885	0.552	0.434	0.925
Clinical + intratumoral + peritumoral radiomics model (ModE)	0.818 (0.731–0.905)	0.677 (0.673–0.682)	0.962	0.567	0.463	0.974

*ACC* Accuracy, *SEN* Sensitivity, *SPE* Specificity, *PPV* Positive Predictive Value, *NPV* Negative Predictive Value

**Table 7:** Delong test of AUC values between different models

Dataset Category	Model	*P*
Training Set	ModA VS ModE	< 0.001
	ModB VS ModE	0.002
	ModC VS ModE	0.002
	ModD VS ModE	0.017
Validation Set	ModA VS ModE	0.109
	ModB VS ModE	0.191
	ModC VS ModE	0.177
	ModD VS ModE	0.395

## Discussion

In this study, five prediction models (ModA, ModB, ModC, ModD, and ModE) were established by combining clinical data with intratumoural and peritumoural factors to predict the occurrence of LVSI in EC. All models achieved good prediction performance; however the AUC of ModE was slightly higher than that of the other four models. The results of this study indicated that ModE can improve the prediction accuracy of the LVSI. Additionally, the nomogram established based on ModE provides a visual tool to enhance the readability of the prediction model and is beneficial for clinicians to evaluate the LVSI in patients before surgery and formulate the best decision-making plan [20, 21].

In our study, the AUC of ModD in the training and validation sets were 0.817 and 0.788, respectively, which were higher than those of ModB and ModC. Our results are consistent with the findings of a previous study, which explored the predictive performance of different radiomics models in predicting LVSI, deep myometrial invasion (DMI) and disease staging of endometrial cancer found that the radiomics models using intratumoral and peritumoral features significantly outperformed the radiomics models using only intratumoral features in terms of predictive performance [22]. Our study also indicated that peritumoural radiomics features possess crucial value in predicting the occurrence of LVSI in EC. This was likely because a transition zone was observed between the tumour and normal tissues, and tumour cells tended to migrate from the primary tumour to the peritumoural area, leading to morphological changes on MRI. Therefore, the peritumoural area contains key information regarding LVSI status [23]. A study predicting lymphovascular invasion in early stage cervical cancer based on the peritumoural radiomics of multiparameter MRI compared the predictive efficacies of peritumoural radiomic features within different scopes. The results showed that the features selected from the peritumoural area with an expansion distance of 3 mm outside the tumour led to the establishment of a model with the best predictive performance [24]. Similarly, in our study, 14 features were extracted from an area with an expansion of 3 mm outside the tumour to establish ModB, and the results demonstrated good predictive performance.

Logistic regression analysis showed that CA125 levels and tumour length were independent clinical risk factors for LVSI in patients with EC. Therefore, a clinical prediction model was established based on the CA125 levels and tumour length. The AUC values of the training and validation sets were 0.805 and 0.766, respectively, indicating that CA125 and tumour length had good predictive values for the presence of LVSI in patients with EC. These findings are consistent with previous studies which have reported that the larger the tumour length, the greater the risk of LVSI in patients [25], while others have shown that elevated CA125 levels can predict positive LVSI in patients with endometrial cancer [26–28].

Notably, the AUC of ModE was 0.870 in the training set, which was higher than AUCs of the other four models (*P* < 0.05). Similarly, the AUC of ModE in the validation set was 0.818, which was higher than those of the other four models (P > 0.05). However, this study has some limitations that require consideration. First, the high AUC values of ModE may have been influenced by the relatively small sample size and imbalance in the baseline data between the training and validation sets [29]. Additionally, the limited sample size may have affected the extraction of imaging features. Therefore, future studies with larger sample sizes are required to validate the results of this study. Second, the intratumoural lesions in our study were delineated manually and were limited by the experience of different physicians in delineating the lesions, which may have led to differences in feature extraction and screening. Subsequent studies are required wherein deep learning is applied to automatically delineate the ROI and improve the generalisation ability of the model.

## Conclusions

This study utilised intratumoural and peritumoural MRI radiomic features combined with clinical indicators to construct a combined clinical + intratumoural + peritumoural radiomic model and nomogram, providing a novel method for accurately predicting the presence of LVSI in patients with EC. This model provides valuable theoretical guidance for preoperative clinical assessments and holds promise for improving patient outcomes.

## Data Availability

The datasets generated and analyzed during the current study are not publicly available due to ongoing analysis and planned future publications. However, they are available from the corresponding author upon reasonable request.

## References

[1] Ferrari F, Giannini A. Approaches to prevention of gynecological malignancies. BMC Womens Health. 2024;24:254. 38654319 10.1186/s12905-024-03100-4 PMC11036672

[2] Maheshwari E, Nougaret S, Stein EB, Rauch GM, Hwang KP, Stafford RJ, et al. Update on MRI in evaluation and treatment of endometrial cancer. Radiographics. 2022;42:2112–30. 36018785 10.1148/rg.220070

[3] Feng J, Lin R, Li H, Wang J, He H. Global and regional trends in the incidence and mortality burden of endometrial cancer, 1990–2019: updated results from the Global Burden of Disease Study, 2019. Chin Med J (Engl). 2024;137:294–302. 37874032 10.1097/CM9.0000000000002841 PMC10836881

[4] Koskas M, Amant F, Mirza MR, Creutzberg CL. Cancer of the corpus uteri: 2021 update. Int J Gynaecol Obstet. 2021;155(Suppl 1):45–60. 34669196 10.1002/ijgo.13866 PMC9297903

[5] Bosse T, Peters EEM, Creutzberg CL, Jürgenliemk-Schulz IM, Jobsen JJ, Mens JWM, et al. Substantial lymph-vascular space invasion (LVSI) is a significant risk factor for recurrence in endometrial cancer–A pooled analysis of PORTEC 1 and 2 trials. Eur J Cancer. 2015;51:1742–50. 26049688 10.1016/j.ejca.2015.05.015

[6] Avesani G, Bonatti M, Venkatesan AM, Nougaret S, Sala E. RadioGraphics update: 2023 FIGO staging system for endometrial cancer. Radiographics. 2024;44: e240084. 38935549 10.1148/rg.240084

[7] Sala E, Rockall A, Kubik-Huch RA. Advances in magnetic resonance imaging of endometrial cancer. Eur Radiol. 2011;21:468–73. 21113597 10.1007/s00330-010-2010-5

[8] Nougaret S, Horta M, Sala E, Lakhman Y, Thomassin-Naggara I, Kido A, et al. Endometrial Cancer MRI staging: updated Guidelines of the European Society of Urogenital Radiology. Eur Radiol. 2019;29:792–805. 29995239 10.1007/s00330-018-5515-y

[9] Arnaiz J, Muñoz AB, Verna V, Gonzalez-Rodilla I, Schneider J. Magnetic resonance imaging for the pre-surgical assessment of endometrial cancer: results in a routine clinical setting, outside dedicated trials; a cross-sectional study. Anticancer Res. 2016;36:1891–4. 27069176

[10] Gillies RJ, Kinahan PE, Hricak H. Radiomics: images are more than pictures, they are data. Radiology. 2016;278:563–77. 26579733 10.1148/radiol.2015151169 PMC4734157

[11] Bitencourt AGV, Gibbs P, Rossi Saccarelli CR, Daimiel I, Lo Gullo R, Fox MJ, et al. MRI-based machine learning radiomics can predict HER2 expression level and pathologic response after neoadjuvant therapy in HER2 overexpressing breast cancer. EBioMedicine. 2020;61: 103042. 33039708 10.1016/j.ebiom.2020.103042 PMC7648120

[12] Yu J, Shi Z, Lian Y, Li Z, Liu T, Gao Y, et al. Noninvasive IDH1 mutation estimation based on a quantitative radiomics approach for grade II glioma. Eur Radiol. 2017;27:3509–22. 28004160 10.1007/s00330-016-4653-3

[13] Jiang X, Song J, Zhang A, Cheng W, Duan S, Liu X, et al. Preoperative assessment of MRI-invisible early-stage endometrial cancer with MRI-based radiomics analysis. J Magn Reson Imaging. 2023;58:247–55. 36259352 10.1002/jmri.28492

[14] Di Donato V, Kontopantelis E, Cuccu I, Sgamba L, Golia D’Augè T, Pernazza A, et al. Magnetic resonance imaging-radiomics in endometrial cancer: a systematic review and meta-analysis. Int J Gynecol Cancer. 2023;33:1070–6. 37094971 10.1136/ijgc-2023-004313

[15] Luo Y, Mei D, Gong J, Zuo M, Guo X. Multiparametric MRI-based radiomics nomogram for predicting lymphovascular space invasion in endometrial carcinoma. J Magn Reson Imaging. 2020;52:1257–62. 32315482 10.1002/jmri.27142

[16] Yan BC, Li Y, Ma FH, Zhang GF, Feng F, Sun MH, et al. Radiologists with MRI-based radiomics aids to predict the pelvic lymph node metastasis in endometrial cancer: a multicenter study. Eur Radiol. 2021;31:411–22. 32749583 10.1007/s00330-020-07099-8

[17] Yan B, Zhao T, Li Z, Ren J, Zhang Y. An MR-based radiomics nomogram including information from the peritumoral region to predict deep myometrial invasion in stage I endometrioid adenocarcinoma: a preliminary study. Br J Radiol. 2023;96: 20230026. 37751166 10.1259/bjr.20230026 PMC10607389

[18] Yan B, Zhao T, Deng Y, Zhang Y. Preoperative prediction of lymph node metastasis in endometrial cancer patients via an intratumoral and peritumoral multiparameter MRI radiomics nomogram. Front Oncol. 2024;14: 1472892. 39364314 10.3389/fonc.2024.1472892 PMC11446724

[19] Lin Z, Wang T, Li Q, Bi Q, Wang Y, Luo Y, et al. Development and validation of MRI-based radiomics model to predict recurrence risk in patients with endometrial cancer: a multicenter study. Eur Radiol. 2023;33:5814–24. 37171486 10.1007/s00330-023-09685-y

[20] Zeng S, Yang P, Xiao S, Liu L. Development and validation of prognostic nomographs for patients with cervical cancer: SEER-based Asian population study. Sci Rep. 2024;14:7681. 38561337 10.1038/s41598-024-57609-7 PMC10984919

[21] Wang Y, Zheng Y, Tian C, Yu J, Rao K, Zeng N, et al. Nomogram based on immune-inflammatory score and classical clinicopathological parameters for predicting the recurrence of endometrial carcinoma: A large, multi-center retrospective study. J Inflamm Res. 2024;17:11437–49. 39735898 10.2147/JIR.S494716 PMC11675361

[22] Yan R, Qin S, Xu J, Zhao W, Xin P, Xing X, et al. A comparison of 2D and 3D magnetic resonance imaging-based intratumoral and peritumoral radiomics models for the prognostic prediction of endometrial cancer: a pilot study. Cancer Imaging. 2024;24:100. 39085930 10.1186/s40644-024-00743-2 PMC11293005

[23] Yan B, Jia Y, Li Z, Ding C, Lu J, Liu J, et al. Preoperative prediction of lymphovascular space invasion in endometrioid adenocarcinoma: an MRI-based radiomics nomogram with consideration of the peritumoral region. Acta Radiol. 2023;64:2636–45. 37312525 10.1177/02841851231181681

[24] Cui L, Yu T, Kan Y, Dong Y, Luo Y, Jiang X. Multi-parametric MRI-based peritumoral radiomics on prediction of lymph-vascular space invasion in early-stage cervical cancer. Diagn Interv Radiol. 2022;28:312–21. 35731710 10.5152/dir.2022.20657 PMC9634933

[25] Akçay A, Gültekin MA, Altıntaş F, Peker AA, Balsak S, Atasoy B, et al. Updated endometrial cancer FIGO staging: the role of MRI in determining newly included histopathological criteria. Abdom Radiol (NY). 2024;49:3711–21. 38836884 10.1007/s00261-024-04398-2

[26] Neilson A, Jamieson A, Chiu D, Leung S, Lum A, Kommoss S, et al. Serum CA125 levels in the context of ProMisE molecular classification provides pre-operative prognostic information that can direct endometrial cancer management. Gynecol Oncol. 2025;193:1–11. 39740305 10.1016/j.ygyno.2024.12.010

[27] Zhou X, Wang H, Wang X. Preoperative CA125 and fibrinogen in patients with endometrial cancer: a risk model for predicting lymphovascular space invasion. J Gynecol Oncol. 2017;28: e11. 27894164 10.3802/jgo.2017.28.e11 PMC5323282

[28] Shawn LyBarger KS, Miller HA, Frieboes HB. CA125 as a predictor of endometrial cancer lymphovascular space invasion and lymph node metastasis for risk stratification in the preoperative setting. Sci Rep. 2022;12:19783. 36396713 10.1038/s41598-022-22026-1 PMC9671890

[29] Li H, Chen XL, Liu H, Liu YS, Li ZL, Pang MH, et al. MRI-based multiregional radiomics for preoperative prediction of tumor deposit and prognosis in resectable rectal cancer: a bicenter study. Eur Radiol. 2023;33:7561–72. 37160427 10.1007/s00330-023-09723-9

